# Distribution of Illness and Medical Expenditure: A Survey in Two Villages in Rural Beijing

**DOI:** 10.1371/journal.pone.0061068

**Published:** 2013-04-02

**Authors:** Jie Song, Hong Ji, Benchang Shia, Shuangge Ma

**Affiliations:** 1 School of Statistics, Capital University of Economics and Business, Beijing, China; 2 Department of Statistics and Information Science, FuJen Catholic University, New TaiPei City, China; 3 School of Public Health, Yale University, New Haven, Connecticut, United States of America; Old Dominion University, United States of America

## Abstract

**Background:**

The main goal of this study is to examine the distributions of illness conditions and resulting medical expenditures and their associated factors. To achieve this goal, an in-house survey was conducted in August of 2012 in rural Beijing, the capital city of China.

**Results:**

The survey was conducted in Nanjianchang and Beijianchang, which are two villages 20 KM away from Miyun, a satellite city of Beijing. Data was collected on 346 households, which included 834 members. Variables measured included household characteristics, household head characteristics, illness conditions, and medical expenditures. Illness conditions and corresponding expenditure were measured for inpatient treatment, outpatient treatment, and self-treatment separately. Multivariate analysis suggested that the presence of inpatient treatment was associated with household head characteristics including age, gender, and education. The presence of a high level of outpatient treatment was associated with household head characteristics including gender and education. The presence of a high level of self-treatment was significantly associated with household size. In the analysis of overall out-of-pocket (OOP) medical expenditure, only age of household head was borderline significant. In the analysis of OOP inpatient expenditure, age and gender of household head were borderline significant. The OOP outpatient expenditure was associated with household size, presence of members older than 60, household head's gender, marital status, and occupation. The OOP self-treatment expenditure was not associated with any household characteristic.

**Conclusions:**

For the surveyed households, medical expenditure made up a considerable proportion of the total consumption. This study suggested that the presence of illness conditions and resulting OOP medical expenditure were associated with certain household and household head characteristics. Such results may help identify the subgroup that is the most affected by illness conditions. As this study collected recent data on inpatient, outpatient, and self-treatment separately, it may provide a useful complement to the existing studies.

## Introduction

In public health studies, illness conditions are important measures for households and their members. Medical expenditure incurred by illness conditions can have profound impact on a household's wellbeing [1], [2], [3], [4]. For example, it has been found that a high level of medical expenditure may lead to a reduced level of basic living expenditure. The distributions of illness conditions and their resulting medical expenditure have been investigated in a large number of studies. See for example [5], [6], [7] and references therein. It has been suggested that such distributions are associated with household characteristics and household head characteristics, although the sets of important factors have been different for different study populations.

China has the world's largest population and the second largest economy by nominal GDP. In the recent years, extensive attention has been devoted to the study of illness conditions, medical expenditure, and health sector and health insurance reform in China. Examples include [8], [9], [10], [11] and many others. Studies have suggested that in the past few years, the health sector in China has experienced a dramatic development. More than 90% of the Chinese population is now covered by the basic health insurance provided by the central and local governments. However, illness conditions remain expensive. It is estimated that the Chinese are paying 40% of health costs themselves as either premiums or out-of-pocket (OOP) payments. Empirical studies have been conducted on the rural-urban difference in the distribution of illness conditions [5], which found that the rural-urban gap was shrinking for central China, whereas the evidence was mixed for western and eastern China. Fang and others conducted a survey study in western China on the distribution of medical expenditure and its associated factors [12], and reported that the level of per capita medical expense was significantly associated with household size, presence of members younger than 18, older than 65, basic health insurance coverage, per capita income, and household head occupation. Chen and others investigated the utility of health services [13] and found that the major influencing factors on the use of healthcare facilities included age, income, health insurance status, previous utilization, and whether on poverty alleviation program. For patients with chronic diseases, Sun and others [14] investigated health insurance coverage and its protection effects, and found that a significant proportion of patients with chronic diseases faced catastrophic healthcare costs, especially for the poor. The NCMS (New Cooperative Medical Insurance Scheme, basic insurance offered in the rural area) offered only a limited degree of financial protection.

The main objective of this study is to examine the distributions of illness conditions and medical expenditures. This objective is similar to that in previous studies such as [2], [5], [6], [7] and others. On the other hand, this study may differ from the published ones along the following directions. In this study, all samples were collected from two villages (with a relatively low financial status) in rural Beijing, while studies such as [11], [12] have focused on large cities and their surrounding areas (where the income levels were considerably higher). China is experiencing fast urbanization, with urban population exceeding countryside for the first time in 2011 [15], [16]. However, as the financial conditions of rural residents were still significantly lower than urban residents, studying rural residents is still of considerable interest [2]. Different from [5] and others, this study investigated inpatient, outpatient, and self-treatments separately, and thus may provide a more comprehensive description of illness conditions and medical expenditure. This study and [2] both focused on rural areas. During the past decade, China has experienced considerable economic growth, with per capita GDP growing at about 10% annually. In addition, the healthcare system has been undergoing system-wide reform. Such changes may have a direct impact on medical expenditure, and may also affect ill health conditions. This study, which is based on data collected in 2012, may have observations different from those in [2].

## Methods

### Data Collection

This study was approved by a research ethics review committee at Capital University of Economics and Business (CUEB), Beijing, China. Data was collected through an in-house survey, which was conducted by research staff at the School of Statistics, CUEB. All samples were collected from two villages, Nanjianchang and Beijianchang, which are located in the Miyun county and about 20 KM from the Miyun City, a satellite city of Beijing. According to census data published by the Miyun county government, in 2010, the two villages combined had 696 households with 1,526 members. Because of financial limitations, the target sample size of this study was 300. The households for survey were randomly selected. A total of 346 households with 834 members participated in this study, with the participation rate (defined as the ratio of the number of participated households and total number of selected households) being 92%. All surveyed subjects were covered by NCMS.

At the beginning of each survey, the staff would introduce the purpose of survey and ask the interviewee to sign a consent form. The written consent forms were stored at CUEB. Basic information was then collected to determine inclusion. By design, a household would be excluded of (1) the household was not officially in the two villages, defined by “Hukou”, a household registration issued by the central government; (2) the interviewee was less than eighteen years old; or (3) the interviewee could not provide reliable information on the household. Less than ten households were excluded because of the aforementioned reasons. The survey included both “snapshot” questions (such as demographic information) as well as “accumulation” questions (such as income and expense over a period of twelve months prior to survey). On average, one survey took less than thirty minutes.

China is a huge country with over 1.3 billion people and economic development and quality of healthcare varying significantly across regions (for example rural versus urban, east versus west). With a limited number of samples, the study results are not expected to be applicable to a large population. Rather, this study may provide useful information for rural populations with similar economic/health status. According to data published by the National Bureau of Statistics of China [17], in 2011, the per capita income of rural residents was 6,977 RMB (compared to 23,979 RMB for urban residents). According to data published by the government of Beijing, in 2010, the per capita income of residents of Miyun was 11,858 RMB (for urban and rural residents combined), and the per capita income of rural residents of Beijing was 13,262 RMB. Summary statistics on income and expense (Table 1) suggested that the samples collected were not at the extreme of distributions.

**Table 1:** Summary statistics: household characteristics for the whole cohort and subgroups with different illness conditions.

		Inpatient		Outpatient		Self-treatment	
	All sample	No	Yes	per person< = 2	per person>2	per person< = 5	per person>5
Sample size	346	280	66	187	159	271	75
Household size Mean (sd)	2.41 (1.057)	2.379 (1.026)	2.545 (1.179)	2.567 (1.092)	2.226 (0.987)	2.557 (1.052)	1.88 (0.900)
Presence of members<18:							
percentage	0.257	0.25	0.288	0.31	0.195	0.299	0.107
Presence of members>60:							
percentage	0.399	0.382	0.47	0.3	0.516	0.332	0.64
Per capita income (RMB):							
mean (sd)	7046 (9869)	7275 (10710)	6077 (4846)	7706 (10827)	6271 (8579)	7641 (10894)	4897 (3897)
Per capita expense (RMB):							
Mean (sd)	11329 (16081)	9948 (15221)	17187 (18303)	10918 (16416)	11814 (15717)	11481 (15697)	10784 (17500)
Per capita medical expense (RMB):							
Mean (sd)	3502 (8187)	1841 (2704)	10546 (16189)	1888.5 (3256)	5399 (11277)	3047 (6204)	5145 (12983)
Per capita inpatient medical expense (RMB):							
Mean (sd)	1662 (7672)	0.000 (0.000)	8713 (15812)	831.9 (2672)	2638 (10878)	1499 (5871)	2250 (12174)
Per capita outpatient medical expense (RMB):							
Mean (sd)	1413 (2546)	1392 (2474)	1504.4 (2852)	576.0 (1598)	2398 (3057)	1273 (2089)	1921 (3737)
Per capita self-treatment expense (RMB):							
Mean (sd)	426 (1010)	449 (1089)	756.8 (563)	480.6 (1130)	645.9 (848)	274.6 (610)	974.5 (1736)
Percentage of medical expense as of total expense:							
mean (sd)	0.349 (0.312)	0.292 (0.293)	0.59 (0.298)	0.251 (0.288)	0.464 (0.309)	0.315 (0.302)	0.472 (0.336)
Percentage of inpatient expense as of total expense:							
mean (sd)	0.085 (0.215)	0.000 (0.000)	0.445 (0.288)	0.071 (0.193)	0.102 (0.238)	0.081 (0.214)	0.098 (0.218)
Percentage of outpatient expense as of total expense:							
mean (sd)	0.184 (0.242)	0.203 (0.253)	0.106 (0.165)	0.085 (0.169)	0.302 (0.261)	0.1812 (0.245)	0.196 (0.233)
Percentage of self-treatment expense as of total expense:							
mean (sd)	0.080 (0.167)	0.089 (0.180)	0.039 (0.079)	0.096 (0.189)	0.061 (0.136)	0.052 (0.127)	0.179 (0.243)

### Statistical Analysis

Data was deidentified prior to analysis. Various graphical methods were employed to examine data, and no outlier was identified. Members in the same households could have correlated illness conditions. In addition in the Chinese tradition, household was the functional unit for major decision making. Income collection and expense distribution were often made at the household level. Thus, household was adopted as the unit for data collection and analysis. Three measurements were adopted to quantify illness conditions, namely the numbers of inpatient treatment, outpatient treatment, and self-treatment. A household member was considered ill if he or she was diagnosed as ill by a healthcare professional, experienced discomfort, or was unable to pursue usual activities. Inpatient treatment was defined as an appointment, procedure and/or treatment requiring an overnight stay in a healthcare facility. Outpatient treatment was defined similarly but without an overnight stay. Outpatient treatment included services and medicine administered by a hospital, community health clinic, private health facility, or village health worker. Self-treatment, also referred to as self-care or self-medication in the literature, was defined as the scenario where an individual used unprescribed drugs or other medical approaches to treat untreated (and often undiagnosed) medical conditions. In the literature, inpatient treatment and outpatient treatment had been studied much more extensively (see for example [11]) that self-treatment.

In analysis, summary statistics on household characteristics and household head (defined unofficially as the person leading decision making) characteristics were first computed for the whole cohort as well as subgroups with different illness conditions. For each type of illness condition (inpatient, outpatient, self-treatment), we contrasted subgroups with a “low” versus a “high” number of episodes (more details provided in the next subsection). Then the associations between illness conditions and household (head) characteristics were examined using multivariate regression. Multivariate analysis was also conducted on medical expense. Here the overall expense was first analyzed, followed by each type of medical expense (inpatient, outpatient, self-treatment) separately. The types of illness conditions for different treatments are usually different, and factors associated with their corresponding expense can be different. Thus, it is sensible to analyze them separately. In the investigation of expense, two sets of analyses were conducted. The first set focused on the actual amount of medical expense (in RMB), whereas the second set studied the percentage of medical expense as of the total per capita expense. Accordingly, linear regression and logistic regression were adopted for the two sets of analyses. Model diagnostics was conducted, and no serious deviation from the model conditions was observed. Analysis was conducted using S-Plus Version 8.2 (TIBCO Software Inc.).

## Results and Discussion

### Household and household head characteristics

Summary statistics on household characteristics and household head characteristics are provided in Table 1 and 2 respectively. As described above, all analyses were conducted at the household level. Among the 346 households, 66 had at least one inpatient treatment. For per-person outpatient treatment, 159 households had more than two. For per-person self-treatment, 75 households had more than five. It is noted that the cutoffs for dichotomization could be somewhat subjective. The intention was to create “high” versus “low” treatment episode groups in a relatively balanced manner (to facilitate downstream logistic regression analysis).

**Table 2:** Summary statistics: household head characteristics for the whole cohort and subgroups with different illness conditions.

		Inpatient		Outpatient		Self-treatment	
	All sample	No	Yes	per person< = 2	per person>2	per person< = 5	per person>5
Age Mean (sd)	57.19 (14.13)	56.63 (13.99)	59.56 (14.61)	54.76 (14.43)	60.04 (13.26)	55.15 (13.73)	64.57 (13.16)
Gender: % of male	0.783	0.768	0.848	0.84	0.283	0.812	0.68
Marital status: %MarriedSingleDivorced/widowed	0.8640.0290.107	0.8640.0320.104	0.8640.0150.121	0.8720.0320.096	0.8550.0250.119	0.8780.0300.092	0.8130.0270.160
Occupation: %FarmerUnemployedOther*	0.7400.0170.243	0.7570.0180.225	0.6670.0150.318	0.7330.0110.257	0.7480.0250.226	0.7310.0110.258	0.7730.0400.187
Education: %No schoolElementary schoolMiddle schoolHigh school and higher	0.2080.3240.3840.084	0.2290.2890.3890.093	0.1210.4700.3640.045	0.2030.2620.4220.112	0.2140.3960.3400.050	0.1730.2990.4430.085	0.3330.4130.1730.080

* Other occupation includes: government, student, self-employed, public or private company and others.

Household characteristics measured included household size, presence of members younger than eighteen, presence of members older than sixty, per capita income for a period of twelve months prior to survey, per capita expense, and per capita medical expense (overall and for each type of treatment, actual amount and percentage as of total expense). Table 1 suggested that a larger household size was associated with more inpatient treatments and fewer outpatient and self-treatments. The presence of members younger than eighteen was also associated with more inpatient treatments and fewer outpatient and self-treatments. The presence of members older than sixty was associated with all three types of treatments. The associations between illness conditions and age distributions had been observed in multiple studies and were intuitively reasonable. Table 1 suggested a negative association between per-capita income and illness conditions. Such an association had been observed and discussed in great details in [6] and others. Table 1 also showed a mismatch between per capita income and expense. A closer examination of data suggested that there were a very small number of households with extremely low income for this particular twelve-month period but with expense levels comparable to other households. That is, the income distribution was skewed. Because of the limited sample size, we were not able to more closely study those households with extremely low income. Although sample mean was perhaps not the most appropriate summary statistic with a skewed distribution, it was chosen because of its simplicity. It should be noted that such mismatch did not necessarily indicate a long-term deficit of the surveyed households, particularly considering the generally high saving rate observed in recent studies conducted in China [11]. Table 1 showed a considerable difference in expense between households with and without inpatient treatment. Difference also existed for outpatient and self-treatments, although the magnitude was much smaller. Such an observation could be partly explained by the differences in financial consequences of different types of treatments [6], [12]. The summary statistics for different types of medical expense, both the actual amount and percentage, were intuitively reasonable. Detailed discussions were omitted.

Household head characteristics collected included age, gender, marital status, occupation and education. Age of household heads was positively associated with the numbers of treatments. An older age is usually correlated with worse health conditions. In the survey, the household sizes were relatively small, which partly explained the observed positive correlation between age of household heads and illness conditions. Although different marital status groups had different illness conditions, such differences did not seem to be dramatic. Differences among different occupation groups were also observed. For example, for households with no inpatient treatment, 75.7% of the household heads were farmers. As a comparison, this number was 66.7% for households with at least one inpatient treatment. There was also a correlation between education and numbers of treatments. For example, for the group with per-person self-treatment less than or equal to five, 17.3% of the household heads had no school. As a comparison, this number was 33.3% for households with per-person self-treatment greater than five.

The above observations were based on marginal analysis. More informative conclusions were drawn below from multivariate analysis.

### Multivariate analysis of illness conditions

The first set of multivariate regression analysis investigated the associations between illness conditions and household/household head characteristics. For each type of treatment, the number of treatment was dichotomized to create a binary response variable. With count data (number of illness episodes), Poisson regression analysis can be potentially more informative, as dichotomization is not needed. However, our preliminary examination suggested that the Poisson assumption might not hold. With binary responses, logistic regression analysis was conducted. The estimated odds ratios and their significance levels were shown in Table 3, where odds ratios greater than one corresponded to positive associations.

**Table 3:** Multivariate logistic regression analysis of illness conditions.

	Inpatient	Outpatient	Self-treatment
**Household characteristics**			
Household size	1.057(0.788)	0.814(0.218)	0.446(0.002)
Presence of members <18	2.009(0.163)	0.966(0.930)	2.014(0.266)
Presence of members >60	0.859(0.775)	2.173(0.062)	1.359(0.555)
Per capita income (1K RMB)	0.992(0.689)	0.978(0.145)	0.944(0.057)
**Household head characteristics**			
Age	1.045(0.041)	1.000(0.983)	1.024(0.239)
Gender (baseline: male)	0.380(0.064)	2.120(0.031)	1.043(0.919)
Marital status (baseline: married)SingleDivorced or widowed	0.724(0.777)2.007(0.239)	0.897(0.881)0.533(0.166)	0.667(0.647)0.563(0.255)
Occupation (baseline: farmer)UnemployedOther*	0.520(0.567)2.034(0.039)	1.789(0.526)1.072(0.805)	3.357(0.196)0.931(0.851)
Education (baseline: no school)Elementary schoolMiddle schoolHigh school and higher	4.397(0.002)2.589(0.091)1.622(0.553)	1.926(0.054)1.796(0.138)1.023(0.968)	1.028(0.940)0.640(0.362)1.501(0.526)

In each cell, odds ratio (p-value). Inpatient: presence of inpatient treatment for a household; Outpatient: per person outpatient treatments>2; Self-treatment: per person self-treatment>5. Other occupation includes: government, student, self-employed, public or private company and others.

Table 3 suggested that household characteristics were not associated with the level of inpatient treatment. Among household head characteristics, age, gender, and education were significantly (or borderline significantly) associated with the presence of inpatient treatment. In particular, age was positively associated (odds ratio 1.045; p-value 0.041). The association for gender was borderline significant, with households with female heads less likely to have inpatient treatment (odds ratio 0.380; p-value 0.064). With no school as baseline, households with heads having elementary school (odds ratio 4.397; p-value 0.002) and middle school (odds ratio 2.589; p-value 0.091) were more likely to have inpatient treatments. However there is a lack of linearity. In the analysis of outpatient treatment, the presence of household members older than sixty had an odds ratio 2.173 (p-value 0.062). Among the household head characteristics, gender was again significant. However, the “direction” was different from inpatient treatment, with household head being female positively associated with the level of outpatient treatment (odds ratio 2.120; p-value 0.031). Among different education groups, the group with elementary school was borderline significantly different from baseline (odds ratio 1.926; p-value 0.054). In the analysis of self-treatment, no household head characteristic was significant. Household size was negatively associated with the level of self-treatment (odds ratio 0.446; p-value 0.002). In addition, per capita income was borderline negatively associated with the level of self-treatment (income measured in 1K RMB, odds ratio 0.944; p-value 0.057).

The associations between illness conditions and household (head) characteristics have been investigated in the literature. See for example [2], [6], [12], [18]. In published studies, the associations between illness conditions and presence of household members older than sixty, per capita income, age of household head, and education of household head have been observed and extensively discussed (arguments will not be repeated here). Findings of this study that may complement the published ones include the association between household size and self-treatment. Compared with inpatient and outpatient, self-treatment has been less investigated [19]. Our literature review suggested that there is a lack of research on the distribution of self-treatment conditions in China. The observed negative association between per capita income and level of self-treatment was consistent with those for inpatient and outpatient treatments (although those two p-values were much larger). The negative association between household size and level of self-treatment was consistent with that for outpatient treatment. Whether it was causal or confounded by other factors warrants further investigation. Another finding was the association between illness conditions and gender of household head, particularly the conflicting signs for inpatient and outpatient treatments. As discussed in [7], [19] and references therein, inpatient and outpatient treatments correspond to significantly different illness conditions and have different characteristics. Our literature search does not render an informative explanation for such observations.

### Multivariate analysis of medical expense

Multivariate analyses of medical expense, both overall and for each type separately, are presented in Table 4 (actual amount in RMB) and 5 (percentage as of total expense) respectively.

**Table 4:** Multivariate linear regression analysis of per capita medical expense.

	Overall	Inpatient	Outpatient	Self-treatment
**Household characteristics**				
Household size	−1008.0(0.118)	−585.9(0.334)	−340.9(0.080)	−81.4(0.308)
Presence of members <18	747.1(0.622)	1129.0(0.428)	−330.1(0.469)	−51.9(0.782)
Presence of members >60	−472.6(0.773)	399.3(0.795)	−1147.0(0.020)	275.4(0.175)
Per capita income (RMB)	−0.019(0.688)	−0.011(0.802)	−0.004(0.761)	−0.004(0.547)
**Household head characteristics**				
Age	116.1(0.072)	102.6(0.090)	18.8(0.333)	−5.4(0.503)
Gender (baseline: male)	−1427.0(0.285)	−2204.0(0.080)	823.6(0.041)	−46.7(0.778)
Marital status (baseline: married)SingleDivorced or widowed	−1373.0(0.628)−1690.0(0.331)	−224.7(0.933)−503.40.758)	−835.6(0.327)−1155.0(0.028)	−313.1(0.372)−31.3(0.884)
Occupation (baseline: farmer)UnemployedOther*	−2656.0(0.442)−715.8(0.515)	−2873.0(0.377)58.1(0.955)	152.3(0.884)−780.1(0.019)	65.0(0.879)6.2(0.964)
Education (baseline:no school)Elementary schoolMiddle schoolHigh school and higher	−199.6(0.880)929.3(0.540)−874.0(0.671)	−365.4(0.768)1415.0(0.322)100.0(0.959)	249.1(0.529)−283.5(0.535)−794.3(0.201)	−83.3(0.609)−202.1(0.283)−180.3(0.480)

In each cell, regression coefficient (p-value).Other occupation includes: government, student, self-employed, public or private company and others.

In the analysis of overall medical expense in Table 4, only the age of household head was borderline significant, with an older age associated with more expense (estimated regression coefficient 116.1; p-value 0.072). Several other factors, although not statistically significant, had large estimated regression coefficients. For example, the regression coefficient for household size was −1008.0 (p-value 0.118), and that for marital status divorced or widowed was −1690.0 (p-value 0.331). Considering that the per capita expense was 11,329 RMB, regression coefficients of such magnitudes may be worth further attention. The lack of significance of these factors can be partly explained by the small sample size and high variation of expense. In the analysis of inpatient medical expense, household head age (estimated regression coefficient 102.6, p-value 0.09) and gender (estimated regression coefficient −2204.0, p-value 0.08) were borderline significant. In the analysis of outpatient expense, household size was borderline significant. Presence of members older than sixty, household head gender, marital status (divorced or widowed) and occupation (other) were significant. In the analysis of self-treatment expense, there was no significant association. There are close connections between the number of illness conditions and expense. Thus as expected, findings in Table 4 are mostly consistent with those in Table 3, with a few exceptions. For example, the significant association between household size and self-treatment observed in Table 3 was no longer significant. This can be partly explained by the relatively small magnitude of self-treatment expense, as can be observed from Table 1.

As discussed in [12] and references therein, the analysis of percentage (of medical expense as of total expense) may better describe the scenario with a fixed budget, since the percentages of all expenses sum to one. Thus to be prudent, the analysis of percentage was conducted and reported in Table 5. The findings were mostly consistent with those in Table 4. Notable differences included that household characteristics were not associated with the percentage of medical expense (for overall and each type separately). In addition, occupation (other, odds ratio 1.86; p-value 0.082) and education (elementary school, odds ratio 3.432; p-value 0.004) were found to be associated with the percentage of inpatient treatment. Published studies such as [12] suggested that different types of expenses (medical, basic, produce, education, saving and investment, etc) were correlated. More detailed data on expenses are needed to fully comprehend the observed associations.

**Table 5:** Multivariate analysis of the percentage of per capita medical expense (as of per capita total expense).

	Overall	Inpatient	Outpatient	Self-treatment
**Household characteristics**				
Household size	0.935(0.731)	1.008(0.968)	1.207(0.381)	0.867(0.478)
Presence of members <18	1.192(0.702)	1.809(0.227)	0.593(0.301)	1.297(0.580)
Presence of members >60	1.177(0.743)	1.069(0.900)	0.878(0.811)	1.100(0.851)
Per capita income (1K RMB)	0.962(0.008)	0.994(0.700)	0.977(0.146)	0.978(0.123)
**Household head characteristics**				
Age	1.061(0.003)	1.043(0.041)	1.026(0.232)	1.023(0.252)
Gender (baseline: male)	1.778(0.156)	0.466(0.077)	2.821(0.020)	1.609(0.252)
Marital status (baseline: married)SingleDivorced or widowed	0.689(0.664)0.547(0.253)	1.161(0.871)1.648(0.374)	0.507(0.471)0.625(0.417)	0.768(0.764)1.234(0.697)
Occupation (baseline: farmer)UnemployedOther*	1.359(0.769)1.127(0.721)	0.475(0.505)1.860(0.082)	3.939(0.234)0.520(0.075)	2.150(0.476)1.517(0.224)
Education (baseline: no school)Elementary schoolMiddle schoolHigh school and higher	1.746(0.162)1.468(0.404)2.381(0.165)	3.432(0.004)2.127(0.125)1.678(0.437)	1.860(0.157)1.180(0.743)1.184(0.806)	1.071(0.867)0.733(0.510)2.074(0.256)

In each cell, odds ratio (p-value). Other occupation includes: government, student, self-employed, public or private company and others.

### Limitations

This study is based on cross-sectional, observational data collected in a survey. Thus, only associations, as opposed to causality, can be inferred. To fully identify factors that cause the variations of illness conditions and medical expenses, more comprehensive data collection or even controlled experiments may be needed. It is noted that many published studies with a similar strategy share the same limitation. Data has been collected using in-house surveys. The nature of survey inevitably leads to certain drawbacks. For example, only a limited number of variables were measured, and it is possible that important factors had been missed in data collection. The set of surveyed variables had been suggested by published studies, and included the most important ones. Interviewees were asked to recall the total amount of expense and income over a period of twelve months. Such an approach may be subject to recall bias [20], especially considering that information on multiple types of illness conditions was collected. Without having access to additional data, such bias cannot be completely ruled out and corrected. Illness conditions were quantified using the numbers of episodes for inpatient, outpatient, and self-treatments. More detailed information, for example the type of disease and the duration of inpatient treatment, may be needed to more comprehensively describe illness. All samples were collected from two villages. With limited resource, focusing on a small area for sampling has been adopted in multiple studies. See for example [2], [3], [6] and others. As described above, the income and expense figures suggested a certain degree of representativeness of the samples. However, it should be noted that the results are only expected to be extendable to regions with similar demographic/financial status.

## Discussion

The study of illness distributions has a long history. Although there is some consistency, the findings in Table 3 do not fully match those made in [2], [5], [6] and others. Multiple factors may have contributed to such differences. In particular, studies such as [3], [6] were conducted in countries other than China, where the financial and cultural environments were significantly different. The studies in [2], [11] investigated rural China. However, the income/expense figures in these studies are significantly different from those in this study, indicating differences in study populations. In addition, with fast economic growth and reform in the health sector, observations made in 2012 (time of our survey) were expected to be different from those made earlier (for example as in [5]).

It was found that multiple factors were associated with medical expense (overall, inpatient and outpatient, but not self-treatment). The findings correlated with but not perfectly matched those with illness distributions. The number of illness conditions has a direct impact on cost. However, many other factors, including type of diseases, duration, health insurance status, and others, also contribute to cost. It was found that multiple characteristics of household heads were significantly associated with medical expense. Household head characteristics were directly associated with her/his health conditions. With an average household size 2.41, one person's (household head) health conditions may have visible associations with a household's medical expense. In addition, household head characteristics may also have an indirect impact on expense by being correlated with other household members' health conditions and affecting households' overall decision making.

### Conclusion

This study has been based on a survey conducted in two villages in rural Beijing. It may provide an update on the illness distribution and associated factors. Because of the significant differences in geographic locations, financial status, and time of data collection, this study has different findings and may complement the existing studies. Another aspect we investigate is medical expense. This study may advance from the published ones by analyzing not only the overall expense but also each type of expense. Different types of treatments correspond to different illness conditions. Thus, results reported in this study can be more comprehensive. Our survey and data analysis may help identify the subgroup that is the most affected by illness conditions and/or has the highest medical expense. Future policy intervention may use that information to identify proper targets.
